# Genetic Differentiation Between Black Grouse (*Lyrurus tetrix*, Linnaeus, 1758) Populations from the Sudetes and the Carpathians

**DOI:** 10.3390/genes17091038

**Published:** 2026-08-29

**Authors:** Anna Santorek, Artur Pałucki, Tomasz Zwijacz-Kozica, Sebastian Szczepański, Michał Ciach, Robert Rutkowski

**Affiliations:** 1 Museum and Institute of Zoology PAS, Twarda 51/55, 00-818 Warsaw, Poland; asantorek@miiz.waw.pl; 2 Karkonosze National Park, Chałubińskiego 23, 58-570 Jelenia Góra, Poland; artur.palucki@kpnmab.pl; 3 Tatra National Park, Kuźnice 1, 34-500 Zakopane, Poland; tzwijacz@tpn.pl; 4 Institute of Plant Genetics, Polish Academy of Sciences, Strzeszyńska 34, 60-479 Poznań, Poland; sszc@igr.poznan.pl; 5 Department of Forest Biodiversity, Faculty of Forestry, University of Agriculture in Krakow, al. 29 Listopada 46, 31-425 Kraków, Poland; michal.ciach@urk.edu.pl

**Keywords:** the black grouse, *Lyrurus* (*Tetrao*) *tetrix*, microsatellite, genotyping, non-invasive sampling, the Sudetes, the Carpathians, genetic differentiation

## Abstract

**Background/Objectives:** The black grouse (*Lyrurus tetrix*) is experiencing a rapid decline across much of Europe. In Poland, the Carpathians and Sudetes constitute two of the last important strongholds of this endangered species; however, the genetic relationships between populations inhabiting these mountain systems remain poorly understood. **Methods:** A total of 579 non-invasive samples were collected in the Jizera Mountains and Giant Mountains (Sudetes), as well as in the Tatra Mountains and the Orava–Nowy Targ Basin (Carpathians). Microsatellite genotyping at nine loci identified 122 individuals. Genetic diversity and population genetic structure were assessed using F-statistics, AMOVA, STRUCTURE, PCoA and DAPC. **Results:** Significant genetic differentiation was detected among populations, with substantially lower differentiation within the mountain regions than between the Sudetes and Carpathians. Analyses consistently identified two major genetic groups corresponding to the Sudetes and Carpathians. The highest genetic diversity was recorded in the Tatra Mountains. Some individuals from this population displayed genetic signatures consistent with possible connectivity to neighbouring populations outside the study area, although this hypothesis requires testing with additional reference samples from these populations. **Conclusions:** The results demonstrate strong genetic structuring of black grouse populations in southern Poland and suggest limited contemporary gene flow between the Sudetes and Carpathians mountain ranges. The observed differentiation supports treating the Sudeten and Carpathian populations as separate Management Units. The Tatra Mountains constitute an important reservoir of genetic diversity and may play a key role in maintaining genetic connectivity within the Carpathian population.

## 1. Introduction

### 1.1. The Importance of Mountain Areas for Biodiversity

Mountains are among the most important hotspots of biodiversity worldwide, supporting numerous rare, endemic, and relict species owing to their environmental heterogeneity and wide range of habitats [1,2,3]. In Europe, mountain regions have also served as refugia during past climatic fluctuations and continue to provide important habitats for many species of conservation concern, despite ongoing environmental change [4,5].

In Central Europe, the Carpathians represent one of the continent’s most significant biodiversity refuges, harbouring extensive natural forests and numerous endemic taxa [6,7,8]. In contrast, the Sudetes Mountains, located northwest of the Moravian Gate, support lower levels of biodiversity and endemism, although they also acted as glacial refugia and remained largely unglaciated during the last glaciation [9,10,11]. Genetic differentiation between the Carpathian and Sudetic populations has been documented in several plant and animal taxa, suggesting limited historical connectivity between these mountain systems [12,13].

Mountain habitats are particularly important for European forest grouse, as some of their last demographically stable populations persist in these regions, which also function as reservoirs of unique genetic diversity [14,15,16,17].

### 1.2. Species Characterisation

The black grouse (*Lyrurus tetrix*) is a widespread species, with a geographical range spanning from the British Isles through Russia to North Korea and from Scandinavia to the Alps [18]. In Central Europe, however, populations are severely fragmented and scattered, and the vast majority have experienced a decline in numbers, particularly in recent decades [19]. The situation in Central and Eastern Europe, including Poland, appears particularly critical, where many populations have become extirpated, and the species is on the brink of extirpation from this region [20,21,22]. Even in regions of Europe considered the core stronghold of the black grouse distribution, such as Fennoscandia, where the species is still hunted, the population is experiencing a moderate but systematic decline [19,23]. The main causes of these declines include changes in the environment and climate, predator pressure, and human disturbance, such as tourism and exploitation of forest resources (summarised in [19]).

Currently, black grouse is considered extremely rare and endangered (EN) in Poland [24]. The decline in numbers became evident in the second half of the 20th century: at the turn of the 1970s and 1980s, it was estimated that the national population decreased from 30,000 individuals to 13,000 (a 60% decline) in less than a decade. In the mid-1990s, it was estimated that the black grouse population was even smaller (approximately 5500 individuals), which equated to a decline of over 70% within a decade [22,25]. Despite the introduction of a hunting ban in 1995, the population continued to decline, and the species disappeared from a significant portion of the area it had occupied until recently [21]. At the beginning of the 21st century, the number of individuals was 2000–2500, but in 2009 only 750 birds were recorded [26,27]. Hence, in the last 50 years, the population has decreased a hundredfold and currently does not exceed 250 individuals, which are scattered across the Sudetes (Karkonosze/the Giant Mountains and Jizera Mountains), the Tatra Mountains, the Orava-Nowy Targ Basin, the Biebrza Valley, and Masuria [22].

### 1.3. The Aim of the Study

The situation of the species in Poland, which requires active conservation measures, triggered genetic studies of the last existing populations [21]. It was found that (i) the black grouse populations occurring in Poland, phylogeographically belong to a large group of populations referred to as the ‘northern *tetrix*’; (ii) at a national scale, there is clear genetic differentiation between geographical distributed populations; (iii) the population from the Jizera Mountains was the most genetically distinct, which suggested that the Sudetes area may be inhabited by a very different genetic group.

The aim of the present study is to expand the sample collection in the ‘northern *tetrix*’ to characterise the population genetics of black grouse in its last refugia. The mountainous Carpathian population, an important stronghold of the species in Poland, has not yet been comprehensively characterised genetically. Previous analyses included only black grouse from the Orava-Nowy Targ Basin, a geographically and environmentally distinct large mountain valley of the Western Carpathians, and therefore contrasting with the Tatra Mountains, which are the highest mountain range in the Carpathians. The results indicated a clear genetic distinctness of the Sudetes population [21]. However, because the inference about the populations in the Sudetes was based on samples from the Jizera Mountains only, we sought to expand the sampling region [21].

## 2. Material and Methods

### 2.1. Sampling

**Figure 1:**
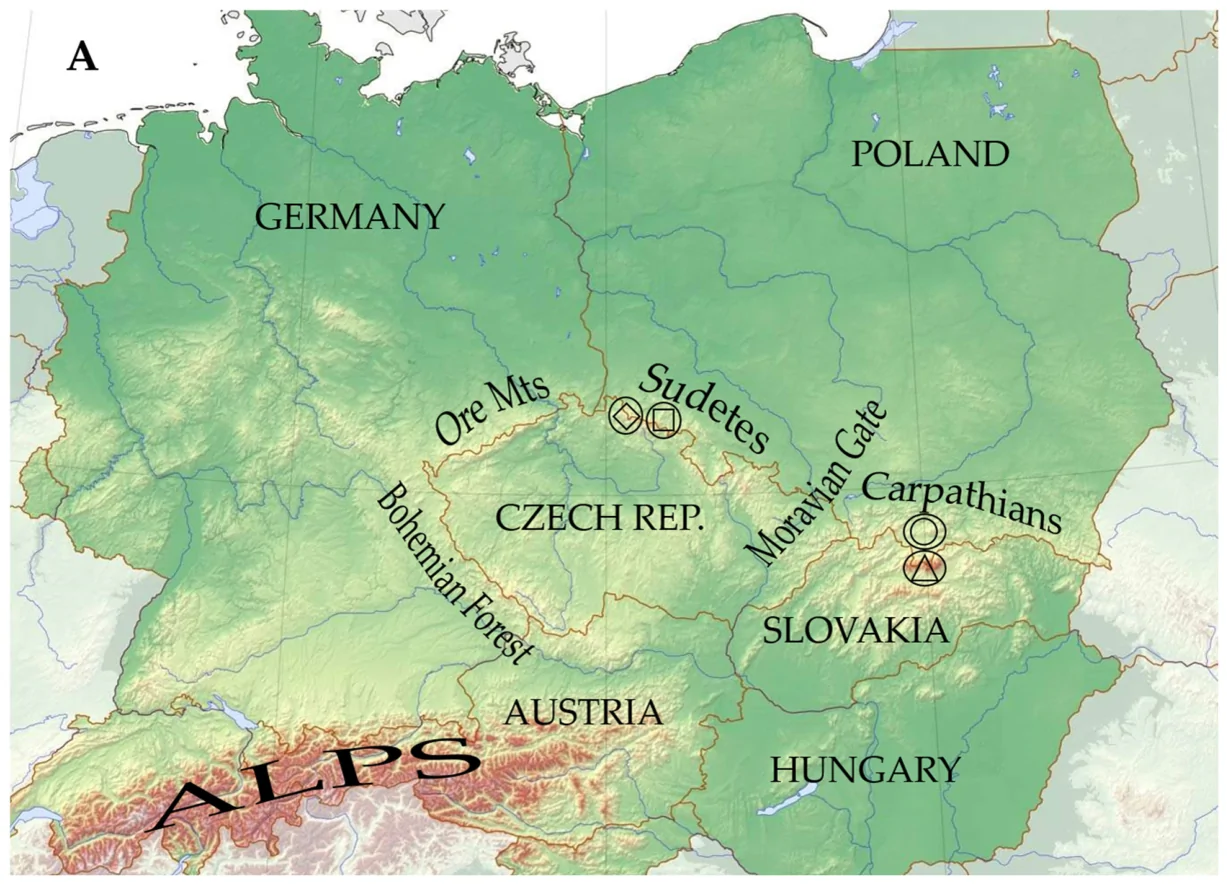

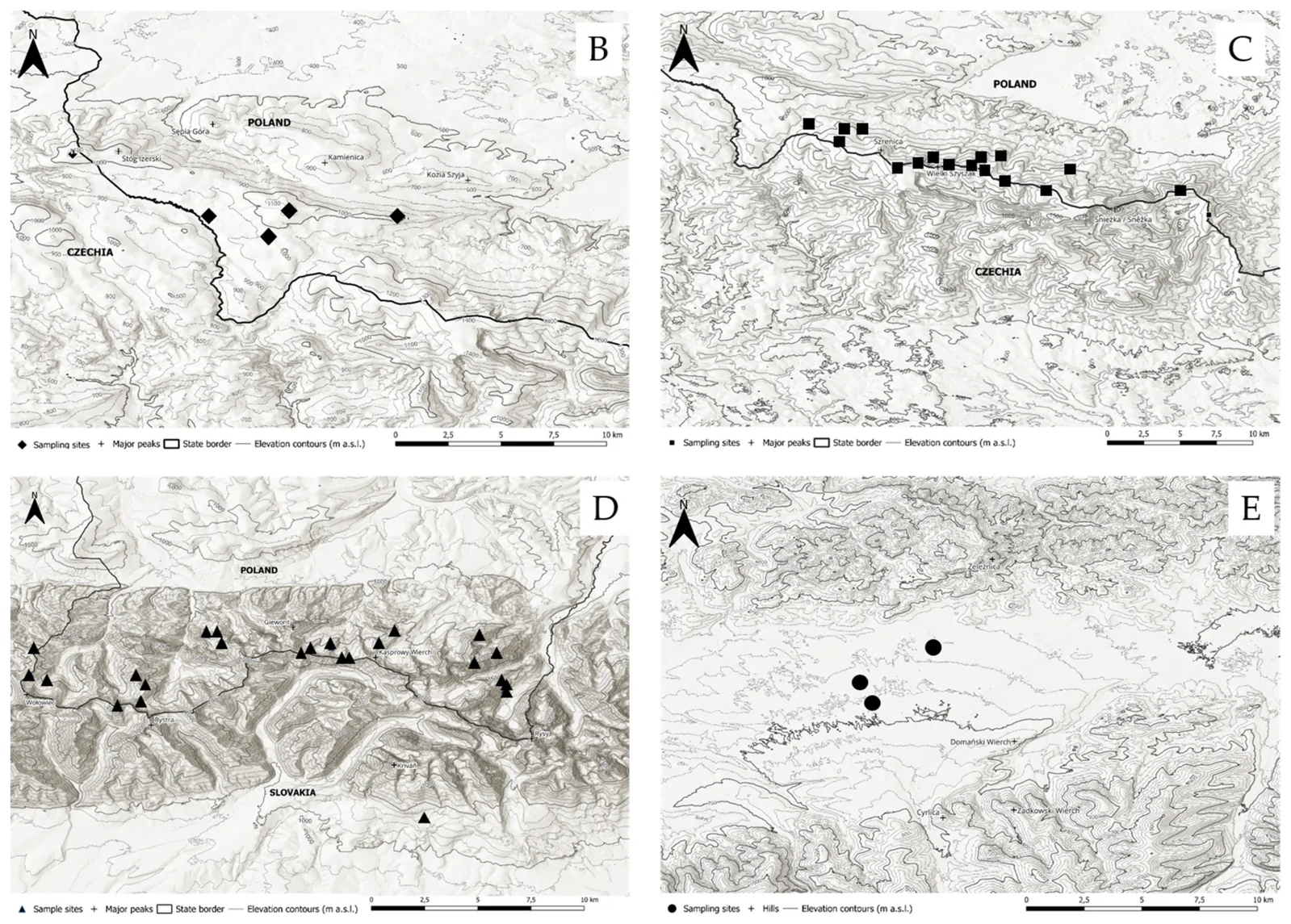
Distribution of sampling sites: (**A**). Investigated populations against the background of the main mountain ranges in Central Europe; (**B**–**E**). Distribution of sampling sites within investigated populations. Diamonds—Jizera Mountains; Squares—The Giant Mountains; Triangles—Tatra Mountains; Circles—Orava-Nowy Targ Basin. Adapted by the authors based on FreeWorldMaps.net (https://www.freeworldmaps.net, accessed on 3 June 2026).

Five hundred and seventy-nine samples were collected between 2009 and 2019 from four mountain ranges: 1) Jizera Mountains (JZM), the Giant Mountains (GM), the Tatra Mountains (TM), and the Orava-Nowy Targ Basin (ONTB) (Figure 1, Table S1). All samples collected in the field were from non-invasive sources (feathers and faeces) and immediately stored in a −20 °C freezer until DNA extraction.

### 2.2. DNA Extraction and Microsatellite Amplification

DNA was isolated from feather samples with the GeneMATRIX Tissue & Bacterial DNA Purification Kit (EURx, Gdańsk, Poland) following the manufacturer’s recommendations for tissue material. Faecal samples were processed using NucleoSpin Soil Kits (Macherey-Nagel GmbH & Co. KG, Düren, Germany), with the modification that the volume of lysate applied to each extraction was doubled. Because all analysed material was obtained non-invasively, additional precautions were implemented to reduce contamination risk and limit genotyping errors, following procedures described previously [16].

DNA samples were amplified at nine tetranucleotide microsatellites previously developed for Tetraonidae species [28,29,30]: TUT1, TUT2, TUT3, TUT4, TTT1, Bg12, Bg15, Bg16, and Bg18.

Three multiplex PCR sets were used. MIX A included primers for Bg16, TTT1, TUT2 and Bg12; MIX B comprised TUT4, TUT3 and Bg18; whereas MIX C contained primers for TUT1 and Bg15. Forward primers were labelled with WellRED fluorescent dyes (Dye2, Dye3 or Dye4) by Sigma-Aldrich (St. Louis, MO, USA). Each 15 μL PCR reaction consisted of 1.5 μL primer mix (2 pmol/μL of each forward and reverse primer), 7.5 μL QIAGEN PCR MasterMix (QIAGEN GmbH, Hilden, Germany) and 1–3 μL DNA extract depending on sample type (1 μL for feathers and 3 μL for faeces). For faecal DNA, 0.3 μL of PCR anti-inhibitor (DNA Gdańsk, currently BLIRT S.A., Gdańsk, Poland) was additionally included. The remaining volume was filled with PCR-grade water (Sigma-Aldrich). Thermal cycling conditions comprised an initial denaturation at 95 °C for 15 min, followed by 40 cycles of 94 °C for 30 s, 57 °C for 90 s and 72 °C for 90 s, a final cycle of 94 °C for 30 s and 57 °C for 90 s, and a terminal extension at 72 °C for 10 min. Fragment analysis was conducted on a CEQ 8000 automated sequencer (Beckman Coulter, Fullerton, CA, USA).

The protocol followed to obtain reliable genotypes was as described by [16]. First, each extract, including a ‘blank test’ for each extraction run (all reagents and procedures, but no DNA), was genotyped three times using the full set of microsatellite markers, and the results were analysed on an automated sequencer. Extracts showing contamination (PCR product in the ‘blank test’ or more than two peaks in the chromatogram at a given locus) were removed from further analysis, and the extractions were repeated. Samples that did not amplify successfully in all three initial replicates were excluded and not considered further. For extracts in which discrepancies among replicates could be attributed to typical artefacts of non-invasive genotyping [31], particularly allelic drop-out (ADO) or false alleles (FA), three additional PCR amplifications were performed. Consensus genotypes were then established using information from all six reactions. Samples showing persistent inconsistencies across replicate amplifications were omitted from subsequent analyses.

### 2.3. Identification of Genotypes and Dealing with Genotyping Errors

Comparisons of genotypes were performed using GenAlEx v. 6.501 [32] based on nine microsatellite loci. When relying on biological material collected noninvasively, it is important to be aware that several or even dozens of independently found samples may originate from the same individual. To assess the reliability of this approach based on genotypic data, for each locus within each population, as well as for a combination of nine loci, Probability of Identity (the average probability that two unrelated individuals randomly sampled from a population will be of the same genotype, *P*_(ID)_) was calculated using GenAlEx. We also calculated the Probability of Identity, considering genetic similarity among siblings (*P*_(ID-Sibs)_). Genotyping error rates were quantified by estimating the frequencies of ADO and FA. Error rates were calculated independently for each locus using genotypes obtained from repeated PCR amplifications of the same DNA extract after comparison with the established consensus genotype. Based on these estimates (see Results), identical microsatellite genotypes observed in two or more independently collected sampling sets were considered to originate from the same individual. Given that, despite the preventive procedures implemented, genotype misidentification may result from genotyping errors, including ADO, FA (as mentioned above), and microsatellite stuttering, the presence of such errors was evaluated using Micro-Checker v.2.2.3 [33]. Even though tests showed that such problems are exceptional (see Results), we assumed that, if differences between genotypes occurred at a maximum of two loci and the discrepancy could be explained by ADO, such genotypes were treated as identical, and the consensus genotype was included in the population analyses. Thus, we obtained unique genotypes for each investigated bird population.

### 2.4. Population Genetics Analysis

Population-level analyses were performed using unique multilocus genotypes identified across the nine microsatellite loci. The occurrence of null alleles was evaluated in R [34] with the package PopGenReport v.3.1.3 [35]. Brookfield’s estimator [36] was applied because it is considered appropriate for datasets lacking missing genotypes, which was the case in the present study. Confidence intervals (95%) were calculated by bootstrap resampling. Deviations from Hardy-Weinberg equilibrium (*HWE*) and linkage disequilibrium (*LD*) were tested separately for each locus and population using Fisher’s exact tests implemented in Genepop v.4.8.5 [37], with 10,000 dememorisations, 1000 batches and 10,000 iterations.

For every population we estimated the mean number of alleles per locus (*A*), allelic richness corrected for unequal sample size by rarefaction to 15 individuals (*R*) [38], the number of private alleles (*P*), observed heterozygosity (*H*_O_), expected heterozygosity (*H*_E_) [39], and the inbreeding coefficient (*F*_IS_). Calculations were carried out using GenAlEx (*A*, *P*, *H*_O_ and *H*_E_), FSTAT version 2.9.3.2 (*F*_IS_) [40], and HP-RARE (*R*) [41].

Population differentiation was quantified using *F*_ST_ [42] under the Infinite Allele Model. Overall and pairwise *F*_ST_ values, together with their significance levels and 95% confidence intervals, were estimated in FSTAT. Significance thresholds were adjusted using Bonferroni correction to account for multiple testing.

To further characterise population structure, an analysis of molecular variance (AMOVA) based on codominant genotypic distances was implemented in GenAlEx. Genetic variance was partitioned into within-population and among-population components. Statistical significance of Φ_PT_ values was assessed using 9999 permutations. Pairwise Φ_PT_ values among populations were evaluated with the same permutation procedure. Relationships among individuals were additionally visualised through Principal Coordinates Analysis (PCoA) using codominant genotypic distances. Analyses were based on a standardised covariance matrix, and the first two coordinate axes were used to illustrate patterns of genetic similarity among sampled birds.

Bayesian clustering analyses were conducted in STRUCTURE v.2.3.4 [43]. For each tested value of *K*, fifteen independent runs were performed using a burn-in period of 100,000 iterations followed by 1,000,000 MCMC iterations. An admixture ancestry model together with correlated allele frequencies was applied. Sampling locations were not incorporated as prior information. The most likely number of clusters was evaluated using the Δ*K* approach [44] and the method proposed by Puechmaille [45]. Output files were processed with StructureSelector and Clumpak [46,47]. The final inference regarding *K* was based on MedMed K, MedMean K, MaxMed K, MaxMean K, Δ*K* statistics, and Mean LnP(*K*) values.

Because STRUCTURE relies on optimisation with respect to Hardy-Weinberg expectations and some sampling locations departed from HWE assumptions, we additionally analysed population structure using Discriminant Analysis of Principal Components (DAPC). This multivariate approach does not depend on Hardy–Weinberg equilibrium and instead relies on genetic variation summarised through principal components and discriminant functions. DAPC was performed in ADEGENET v.1.3.4 [48]. Principal component analysis was used as a preliminary step, followed by discriminant analysis for cluster identification [49]. Genetic relationships among individuals were visualised using the first two discriminant axes ranked according to their eigenvalues.

## 3. Results

### 3.1. Microsatellite Amplification, Genotype Identification and Genotyping Errors

Of the 579 samples, 378 (ca. 65% of the total number collected) were included in the final analysis. One hundred and twenty-two samples (21% of the total number collected) failed to amplify, and 79 additional samples failed to yield consistent results over the six PCRs.

The microsatellite genotyping error analysis demonstrated a high level of reliability of the obtained genetic data. Across all nine loci, the overall genotyping error rate was low, reaching only 4.30%, with allelic drop-out (ADO) accounting for 3.60% of successful amplifications and false alleles (FA) representing only 0.70% (Table S2). The per-locus Probability of Identity (*P*_(ID)_) ranged from 0.61 (TUT2 in JZM) to 0.44 (Bg12, TM). For the combination of 9 loci, all *P*_(ID)_ values were lower than 2.2 × 10^−7^, while *P*_(ID-Sibs)_ was lower than 8.4 × 10^−4^. Hence, according to our data, the expected number of different individuals with the same genotype was very low. Micro-Checker did not suggest any problems with either stuttering or large-allele drop-outs. Considering the results presented above, identical genotypes obtained from non-invasive samples, as well as highly similar genotypes (as defined in the Methods, i.e., differing by no more than two alleles, with such discrepancies attributable to allelic drop-out [ADO] events), were assumed to originate from the same individual. Identical multilocus genotypes were detected in both faeces and feathers. The sample size for analysis was reduced accordingly, with 22 distinct individuals sampled from the Jizera Mountains (JZM), 43 from the Giant Mountains (GM), 42 from the Tatras (TM), and 15 from the Orava-Nowy Targ Basin (ONTB).

### 3.2. Population Genetics Analysis

#### 3.2.1. Null Alleles and Linkage Disequilibrium

The method of Brookfield [36] indicated the possibility of null alleles for three loci: TTT1, TUT1, and Bg18 (frequency below 10%), but only in the case of the pooled dataset. Analysis of particular populations failed to indicate null alleles.

Among the 36 locus × locus combinations, three cases (≈8%) of significant linkage disequilibrium were noted for the investigated loci, following Bonferroni correction (adjusted *p*-value for the 5% nominal level = 0.001389, 720 permutations). This concerned loci Bg16, TTT1, Bg16, Bg12, and TTT1, and Bg18, but only in the case of the Tatra population. Because this *LD* did not appear consistently among populations and was not detected in a previous study [16], we assumed that the signs of linkage could be attributed to the specific characteristics of TM rather than the physical linkage between the markers.

#### 3.2.2. Genetic Diversity

Overall, all microsatellites were polymorphic, with 3 (TUT2) to 14 (Bg12) alleles per locus. However, when analysing the results by population, the TUT2 locus was found to be monomorphic in the Giant Mountains. On average, almost eight alleles were identified per locus, and the observed heterozygosity exceeded 0.6 (Table 1, Table S3). Analysis of all individuals as a single group revealed no deviation in heterozygosity from the Hardy–Weinberg equilibrium. However, the overall inbreeding coefficient (*F*_IS_) was positive and significant after Bonferroni correction, indicating a deficiency in the observed number of heterozygotes compared with the expected value (Table 1). The individual populations were in *HWE*. The inbreeding coefficient was low and not significantly different from zero for each population. The number of alleles (allelic diversity, *A*) found in the studied bird groups was clearly related to the sample size and ranged from 4.5 to 5.8. Comparison of the indicator, taking into account differences in sample size (allelic richness, *R*), indicated that the highest genetic variability can be attributed to the Tatra population (>5.0), while the remaining studied populations have a similar allelic richness at the level of 4.3 to 4.6. The highest heterozygosity and number of private alleles (*P*) were also found in the TM population (Table 1). In Table 2, we summarise the genetic diversity indices estimated from microsatellite markers in European black grouse populations studied to date.

#### 3.2.3. Genetic Differentiation

We found significant genetic differentiation among the investigated populations [overall *F*_ST_ = 0.153 (95%CI 0.096–0.215)]. All pairwise *F*_ST_ comparisons were significant (Bonferroni correction, corrected *p*-value = 0.008, 120 permutations). Genetic differentiation within Sudetes (JZM, GM *F*_ST_ = 0.0265) was clearly lower than between each Sudetes location and TM and ONTB (GM, ONTB *F*_ST_ = 0.2242; GM, TM *F*_ST_ = 0.1846; JZM, ONTB *F*_ST_ = 0.2170; JZM, TM *F*_ST_ = 0.169). Accordingly, genetic differentiation between TM and ONTB was low (*F*_ST_ = 0.0767), but higher than in the case of Sudetes locations (*F*_ST_ = 0.0265). Nonetheless, we observed low genetic differentiation within geographical regions and much higher values for comparisons among locations from different regions. This pattern was confirmed by AMOVA, which showed significant genetic differentiation among the studied populations (Φ_PT_ = 0.263; *p* < 0.001), with as much as 26% of the variation attributed to differences between geographical regions (*p* < 0.001).

**Figure 2:**
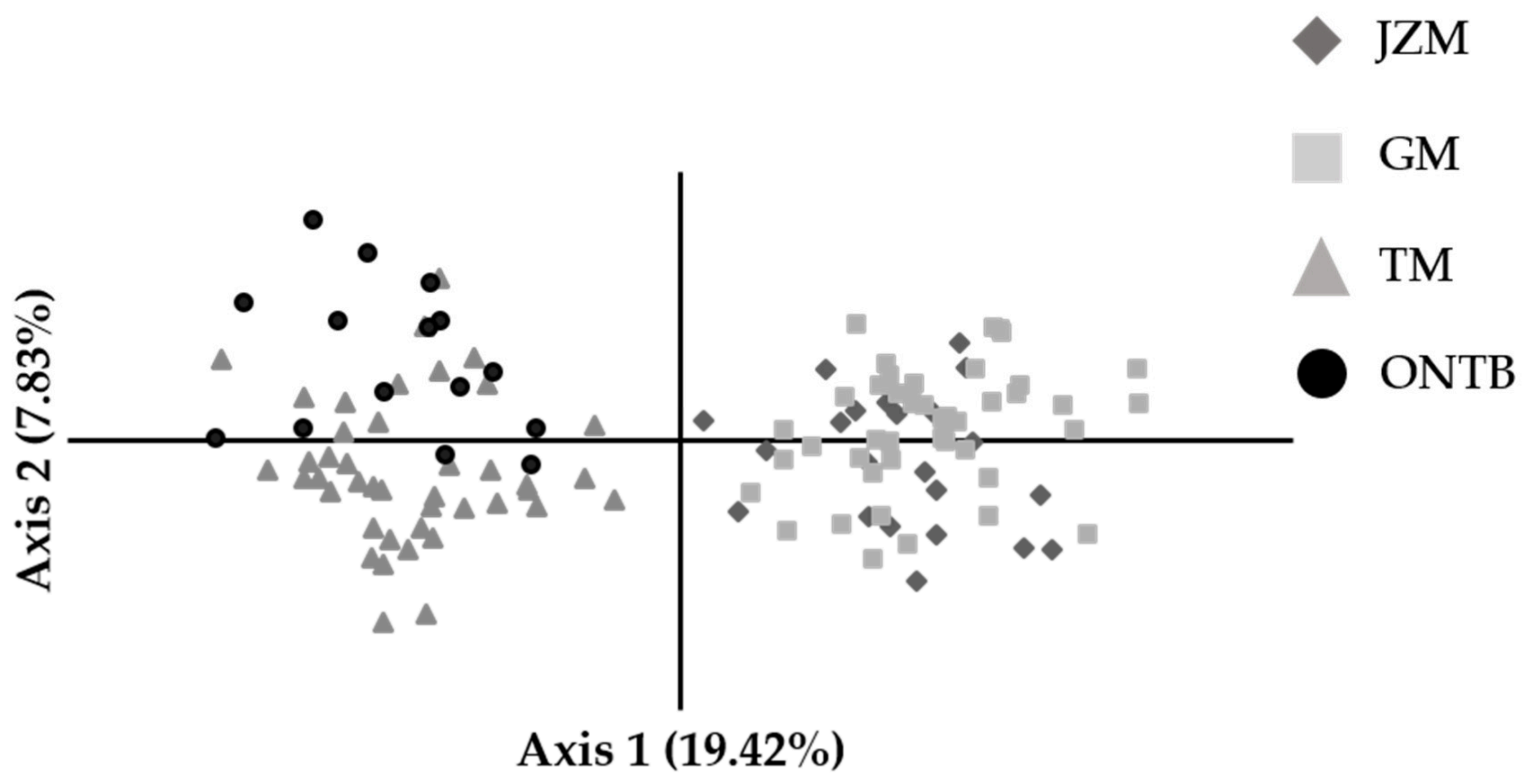
Principal Coordinates Analysis (PCoA) based on multilocus microsatellite genotypes of black grouse (*Lyrurus tetrix*) individuals from four populations in Poland, performed in GenAlEx. Symbols denote populations: JZM (the Jizera Mountains, diamonds), GM (the Giant Mountains, squares), TM (Tatra Mountains, triangles), and ONTB (Orava–Nowy Targ Basin, circles). The first two axes explained 19.42% and 7.83% of the total genetic variation, respectively.

Principal coordinate analysis (PCoA) supported clear genetic differentiation among mountain ranges (Sudetes vs. Carpathians). The first two principal coordinates explained 27.24% of the total genetic variation, with the first and second axes accounting for 19.42% and 7.83% of the variation, respectively (Figure 2). The ordination plot showed that individuals from the JZM and GM largely overlapped in the PCoA space, indicating a close genetic relationship between these populations. Birds from the TM and ONTB also formed a common genetic group on the negative side of the first PCoA axis, indicating a close genetic relationship between these two populations. However, some degree of differentiation between these two Carpathian populations was observed along Axis 2.

**Figure 3:**
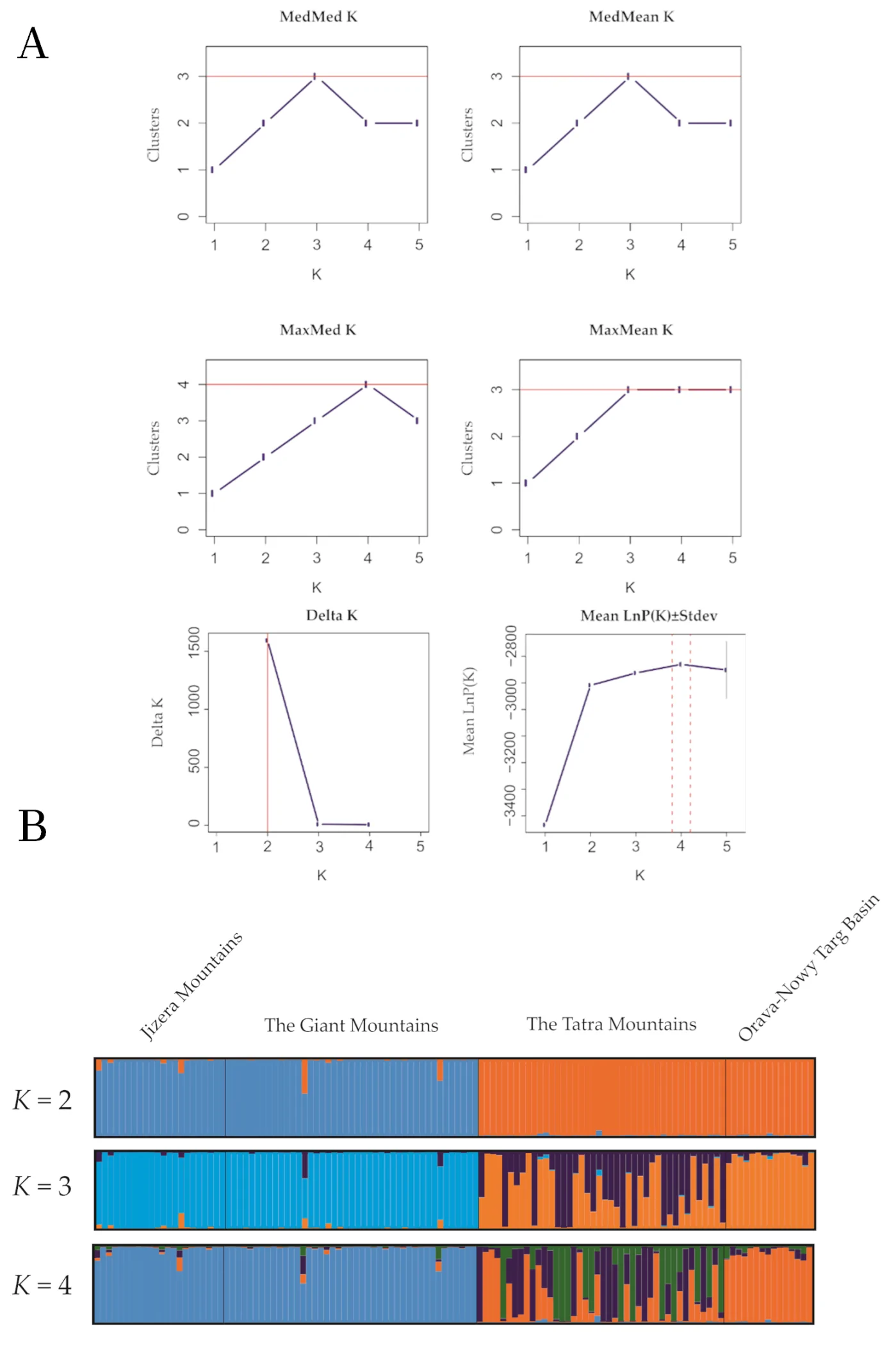
(**A**). Graphical summary of the analysis of the results obtained in STRUCTURE software: MedMed K—Median of medians, MedMean K—Median of means, Max-Med K—Maximum of medians and MaxMean K—Maximum of means [57]; Delta K as described by [56]; and Mean LnP(K)—maximum likelihood and the standard deviation (±Stdev) [55], based on genotypes in 9 microsatellite loci of 122 individuals of the black grouse (*L. tetrix*). The red horizontal lines represent the maximum value of each clustering validation index. The red vertical dashed lines indicated the *K* values corresponding to the highest Mean LnP(K). (**B**). Results of analysis in STRUCTURE software for different values of inferred genetic clusters (*K*), based on genotypes in 9 microsatellite loci of 122 individuals of the black grouse (*L. tetrix*). In bar plots, each individual is represented by a vertical bar partitioned into segments. The length of each segment describes the estimated membership proportions characterising each of the genetic clusters.

The following patterns of differentiation were supported by STRUCTURE analyses, although different clustering methods indicated a range of plausible solutions from *K* = 2 to *K* = 4. The Δ*K* method identified two main genetic groups corresponding broadly to individuals from the Sudetes and Carpathian regions (Figure 3A,B). Most median-based estimators supported *K* = 3, in which individuals from the Giant Mountains and Jizera Mountains formed a relatively homogeneous genetic cluster, whereas birds from the Tatra Mountains and the Orava-Nowy Targ Basin showed partial assignment to two genetic clusters. In this scenario, one cluster predominated in the Orava-Nowy Targ Basin, whereas individuals from the Tatra Mountains exhibited mixed assignment proportions (Figure 3A,B). The *K* = 4 solution, supported by Max-Med *K* and Mean LnP(*K*), indicated relatively homogeneous genetic groups in the Sudetes and the Orava-Nowy Targ Basin, whereas individuals from the Tatra Mountains displayed a higher degree of admixture and assignment to multiple clusters (Figure 3A,B).

**Figure 4:**
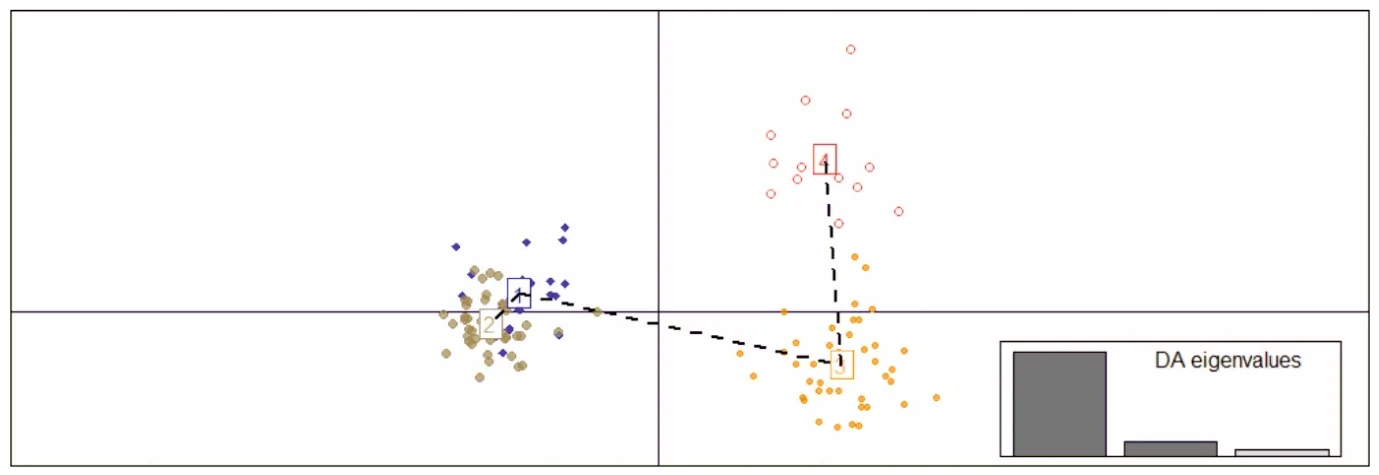
DAPC scatter plot of 122 individuals of the black grouse (*L. tetrix*), based on multilocus microsatellite genotypes: 1 [blue squares]—Jizera Mountains; 2 [grey squares]—The Giant Mountains; 3 [solid yellow circles]—The Tatra Mountains; 4 [empty red circles]—Orava-Nowy Targ Basin.

The DAPC results were consistent with the STRUCTURE analyses and supported the inferred pattern of genetic clustering in the study. Cross-validation was conducted using 2–20 retained principal components, and the highest mean assignment success was achieved with 12 PCs (69.7%). Therefore, 12 PCs and three discriminant functions were retained for final analysis. The first two discriminant axes clearly separated individuals from the Sudetes and Carpathian regions, whereas birds from the Jizera and Giant Mountains largely overlapped in multivariate space. Individuals from the Tatra Mountains and Orava-Nowy Targ Basin showed substantial overlap, although partial separation between these two sampling locations was also apparent (Figure 4).

## 4. Discussion

We detected 122 black grouse individuals in Poland over a 10-year period. The current population of the species in Poland is unknown due to the lack of uniform, nationwide monitoring [27]. Until recently, approximately 50–65 males (≈100–130 individuals) probably occurred in the Tatra Mountains and 30–40 males in Orava-Nowy Targ Basin [27,57], while in the Sudetes, the estimated population was much higher, with approximately 120 males combined in the Jizera and Giant Mountains. The primary objective of this study was not to estimate population size but to assess genetic diversity and genetic differentiation between the two major mountain strongholds of the species in southern Poland. Reliable population size estimation requires a dedicated sampling design in both space and time and cannot be inferred directly from the number of genotyped individuals obtained in a genetic survey [58,59]. Moreover, the number of detected individuals strongly depends on the sampling effort, sample availability, and detection probability. Therefore, direct comparisons between the number of identified genotypes and census population estimates should be interpreted with caution. However, the obtained results suggest that the Giant Mountains population may be overestimated or that it is a population experiencing a decline; despite very intensive sampling, a very similar number of individuals was found there as in the Tatra Mountains (*N*_IND_ 65 vs. 57 in the Sudetes and Carpathians, respectively).

All analyses consistently supported substantial differentiation between the populations from the Sudetes and Carpathians. The overall level of genetic differentiation was high, and pairwise comparisons showed significantly lower differentiation within mountain regions than between them. A significant heterozygote deficiency, manifested by a positive and significant *F*_IS_ value, was observed for the overall analysis and, most probably, could be interlinked to limited gene flow between the studied local populations (Wahlund effect), as supported by high and significant *F*_ST_, particularly for comparisons between the two main mountain ranges. This pattern was further supported by AMOVA, PCoA, STRUCTURE, and DAPC analyses. Individuals from the Jizera and Giant Mountains formed a common genetic cluster, whereas birds from the Tatra Mountains and Orava-Nowy Targ Basin constituted a separate Carpathian genetic group. The observed pattern indicates that gene flow is currently much stronger within mountain regions than it is between them. Particularly low differentiation between the Jizera and Giant Mountains (*F*_ST_ = 0.0265) suggests recent or ongoing genetic exchange, whereas differentiation between the Tatra Mountains and Orava-Nowy Targ Basin (*F*_ST_ = 0.0767), although still relatively low, indicates more restricted connectivity. In contrast, all comparisons between the Sudetes and Carpathian populations showed high differentiation (*F*_ST_ = 0.169–0.224), demonstrating the long-term limitation of gene flow between these regions. The distribution of private alleles provides additional insights into population connectivity. The Tatras population contained the largest number of unique allelic variants, whereas fewer private alleles were detected in the remaining populations. The presence of multiple private alleles indicates the long-term persistence of locally unique genetic variation and suggests that effective gene flow between the Carpathians and Sudetes has been limited for a considerable period. If contemporary migration between regions were frequent, many of these alleles would be expected to be shared among populations. Thus, the observed distribution of unique alleles strongly supports the existence of two partially independent genetic lineages in southern Poland. Simultaneously, the low differentiation and shared genetic composition within the Sudetes, as well as the substantial overlap between the Tatra Mountains and Orava-Nowy Targ Basin, demonstrate that functional connectivity still exists at regional scales. Despite their different geological origins, the Carpathians and Sudetes share similar climatic conditions and many endemic species. Therefore, the pronounced genetic differentiation observed between the Sudetes and Carpathians may reflect several non-exclusive processes. These include contemporary and historical limitations to gene flow associated with habitat fragmentation, as well as deeper phylogeographic processes that may have shaped the evolutionary history of the populations inhabiting the two mountain systems. However, the present study was not designed to directly test the mechanisms responsible for these patterns.

Studies in Czechia have shown that genetic differentiation between separate mountain ranges, including the Giant Mountains (Krkonoše) and Jizera Mountains, is significant and relatively high, with many pairwise *F*_ST_ values for local populations even above 0.15–0.18 [50]. This study also showed that the Jizera Mountains form a common genetic group with Krkonoše, while individuals from two other mountain ranges (Ore Mountains [Krušné] and Bohemian Forest [Šumava Mountains]) were assigned to two different clades. Therefore, in the Western Sudetes region, a clear division into genetic groups corresponding to the distribution of mountain ranges is a typical pattern determining the genetic structure of black grouse populations. In other taxa, including the fire salamander (*Salamandra salamandra*), genetic differentiation between Sudeten and Carpathian populations has been interpreted as being associated with the Moravian Gate, a densely populated and highly transformed region separating the two mountain systems [13]. Consequently, the Moravian Gate may represent one of several landscape features potentially contributing to the restricted gene flow between black grouse populations of the Sudetes and Carpathians. However, dedicated landscape genetic analyses are required to directly evaluate its role.

The observed differences between the Sudetes and Carpathian populations may be rooted more deeply than recent population fragmentation, as suggested for some plants [60,61]. Previous studies [21] suggested genetic separation of the Sudetes from other black grouse populations in Poland but did not confirm the north (lowland populations) vs. south (Carpathian populations) division observed for other grouse species in Poland [16,62]. Previously, the ONTB population from the Carpathian region was included in the ‘lowland’ clade, whereas the current study suggests that the ONTB is genetically similar to the Tatra population. It is possible that the black grouse is divided into eastern and western genetic groups. A similar division has been suggested for the hazel grouse (*Tetrastes bonasia*), with the Sudetes population classified as a separate subspecies [62,63]. On the other hand, mitochondrial DNA of birds from the Jizera Mountains and the Giant Mountains has shown that the Sudetes population, in terms of haplotype frequency, is a typical representative of the so-called ‘northern *tetrix*’ evolutionary lineage [21,64], widely distributed in northern and eastern Europe, including the Carpathians. It therefore appears that the black grouse has undergone a similar pattern of genetic differentiation as the fire salamander. The Sudetes populations most likely originated from the Carpathian refugium, as reflected in their mtDNA haplotypes, but the recent isolation of populations from both mountain ranges has led to nuclear DNA differentiation [13].

The obtained results suggest genetic similarity between Carpathian birds (Tatra Mountains) and individuals inhabiting the nearby region of Orava-Nowy Targ Basin. However, this is not a uniform gene pool, as is the case with the two Sudetes locations in Poland. Some analyses indicate that the Tatra Mountains and ONTB share a significant amount of genetic variation, but some individuals from the Tatra Mountains originate from a distinct genetic clade, showing partial assignment to distinct genetic clusters. One possible explanation is connectivity with neighbouring Slovak populations, which are known to occur in the transboundary Tatra region. However, because no Slovak reference samples were included in the present study, this interpretation should be regarded as a hypothesis that requires verification with genetic data from Slovakia.

The highest genetic diversity was recorded in the Tatra Mountains, which exhibited the highest allelic richness (*R* = 5.08), observed heterozygosity (*H*_O_ = 0.66), and number of private alleles. This pattern is consistent with the broader biogeographic importance of the Carpathians, which have been proposed as a refugium for numerous taxa [21,62,65]. However, the microsatellite data analysed in this study do not allow for direct inferences about historical refugial processes. Testing whether the observed diversity reflects a legacy of long-term persistence in the Carpathian refugium would require broader phylogeographic sampling and additional mitochondrial or genomic data.

The lowest genetic variability was found in the Jizera Mountains, consistent with the results of other studies [21,50]. It seems that this local group of black grouse is very small and, therefore, vulnerable to loss of genetic variability and an increase in the level of homozygosity, although at least until recently it was connected by gene flow with the Giant Mountains. Importantly, inbreeding coefficients within populations remained low and non-significant, suggesting that genetic diversity is effectively maintained despite substantial population fragmentation. In the case of ONTB, the *F*_IS_ was negative (although insignificant after Bonferroni correction). It is likely that, at least in this population, the excess of heterozygotes may be a result of the rapid decline in population size in recent years [57]. During a rapid decline in the number of individuals in a population, allelic variants are eliminated, which reduces the genetic diversity estimated from the number of alleles present. Heterozygosity also decreases, but at a much slower rate. Therefore, a situation may arise in which the observed number of alleles is lower than the number of alleles expected in genetic equilibrium, resulting in the periodic detection of a deviation in the form of an excess of heterozygotes [66,67].

A comparison with other European populations demonstrates that the levels of genetic diversity observed in southern Poland are generally comparable to those reported for many mountain populations across the continent (Table 2). Genetic diversity estimates indicate that the studied populations retain moderate to relatively high levels of variation, despite their fragmented distribution. The Tatras population exhibited allelic richness similar to that found in majority of Alpine and historical Central European populations (*R* ≈ 4.8–6.4, [51,52,53]) and higher than values reported for several isolated mountain populations from the Czechia (*R* = 3.3–4.4 [50]), the Netherlands (*R* = 3.6–4.0 [52,53]), Denmark (*R* = 3.9 [52]), and contemporary lowland populations from northern Germany (*R* = 4.5 [52]). Although diversity observed in the Tatras remains lower than in large continuous populations from Finland and Norway (*R* = 5.9–6.5; *H*_O_ = 0.72–0.88 [30,51,53]), it exceeds that reported for many isolated populations that have experienced severe demographic declines [21,52,53]. Similarly, the Sudetes populations display genetic diversity comparable to other isolated mountain populations in Central Europe and are substantially higher than those reported for several declining lowland populations.

Compared to other European studies, the genetic structure detected in southern Poland appears unusually pronounced. While more pronounced genetic differentiation relative to Scandinavian populations was expected due to extensive habitat connectivity and larger effective population sizes, we also detected a substantially stronger genetic structure than that reported for the Alpine metapopulation [54]. In the Alps, gene flow was most likely maintained according to the ‘stepping-stone’ model and was possible due to the existence of a dense network of local subpopulations. It is believed that black grouse fly distances from 5 to 29 km in the case of females, which are the more dispersive sex, and even up to 8.5 km for males [68,69]. Black grouse move along suitable habitats, even for distances of 20 km, and cross valleys by traversing low mountain passes [68]. Apparently, the development of industry, intensive agriculture and urbanisation around the Sudetes and Carpathians, together with the resulting environmental transformations, have led to such drastic habitat loss that the ‘stepping-stone’ model has ceased to function [54,56,70]. In contrast, the differentiation observed between the Sudetes and Carpathians is comparable to or exceeds the values reported among isolated mountain ranges in the Czech Republic [50]. These results indicate that the Polish mountainous populations represent two well-defined genetic units separated by limited contemporary gene flow.

## 5. Conclusions

We documented the distribution of genetic diversity in two major black grouse strongholds in southern Poland. Populations inhabiting the Sudetes and Carpathians represent two clearly differentiated genetic groups. The processes underlying this differentiation remain to be fully resolved. Our results are consistent with restricted contemporary gene flow between mountain regions and may reflect the influence of heavily transformed lowland areas separating them, including the Moravian Gate. However, testing this hypothesis will require dedicated landscape-genetic analyses. The Tatra Mountains contained the highest level of genetic diversity among the studied populations. Some individuals also displayed genetic signatures that may indicate connectivity with neighbouring Slovak populations, but this interpretation requires confirmation using reference samples from Slovakia. Likewise, although the high diversity observed in the Tatras is consistent with the historical importance of the Carpathians as a refugium, the present dataset does not allow direct inference about phylogeographic history. From a conservation perspective, the observed differentiation supports consideration of the Sudetes and Carpathian populations as separate Management Units. The results additionally highlight the importance of maintaining functional landscape connectivity for the long-term persistence of fragmented black grouse populations.

## Figures and Tables

**Table 1:** Summary of sample sizes and allelic diversity and heterozygosity indices at 9 microsatellite loci present among the investigated black grouse (*L. tetrix*) populations. *N*_COL_—Number of samples collected; *N*_GEN_—Number of samples successfully genotyped; *N_IND_*—Number of individuals identified based on unique genotypes; *A*—Mean number of alleles per locus; *R*—Mean allelic richness; *P*—Mean number of private alleles; *H*_O_—Heterozygosity observed; *H*_E_—Heterozygosity expected; *HWE*—*p*-values for *HWE* exact test for heterozygote deficiency/excess; *F*_IS_—Inbreeding coefficient (only overall *F*_IS_ value was significant after Bonferroni correction [*], 720 randomisations, adjusted *p*-value = 0.0014).

	JZM	GM	TM	ONTB	Overall
*N*_COL_	33	347	166	33	579
*N*_GEN_	28	222	104	24	378
*N*_IND_	22	43	42	15	122
*A*	4.65	5.67	5.78	4.56	7.78
*R*	4.26	4.66	5.08	4.55	—
*P*	0.00	0.44	0.56	0.22	—
*H*_O_	0.551	0.599	0.661	0.630	0.616
*H*_E_	0.591	0.593	0.658	0.581	0.702
*HWE*	0.083	0.881	0.673	0.978	0.265
*F*_IS_	0.092	0.001	0.007	−0.049	0.127 *

Statistical and genetic parameters symbols are presented in italics.

**Table 2:** Genetic diversity of the European populations of black grouse (*L. tetrix*), estimated based on allelic richness (*R*) and observed heterozygosity (*H*_O_). Pop.—Geographic location and country where biological samples were collected; Desc.—Ecological and population description of the focal population, based on available demographic and ecological information; Samp. period—Time period during which biological material was collected for genetic analyses; Pop. size—Estimated census population size; Ref.—Reference to the original source publication.

Pop.	Desc.	Samp. Period	Pop. Size	*R*	*H*_O_	Ref.
Orawa-Nowy Targ Basin, Poland	submountain, isolated, decreasing	2012–2016	30–40 birds during the study period	4.55	0.63	This study
Tatra Mts., Poland	mountain	2018–2019	50–65 males (≈100–130 individuals)	5.08	0.66	This study
Sudetes, Poland	mountain, isolated	2009–2018	120 males (≈200–250 individuals)	4.26–4.66	0.55–0.66	This study
Sudetes [Giant Mts. and Jizera Mts.], Poland	mountain, isolated	2009–2015	120 birds during the study period	4.29	0.58	[21]
Orava-Nowy Targ Basin, Poland	submountain, isolated, decreasing	2011–2015	30–40 birds during the study period	4.20	0.66	[21]
North-eastern Poland	lowland, fragmented, decreasing	2013–2015	>100 birds	3.38–5.53	0.53–070	[21]
Poland	introduced	2009, 2012	–	5.22–5.75	0.74–0.82	[21]
Šumava [Bohemian Forest] Czech Rep.	mountain, isolated	2004–2007	80 males in 2005	3.53–4.44	0.57–0.74	[50]
Krkonoše [Giant Mts.], Czech Rep.	mountain, isolated	2004–2007	128 males in 2005	3.33–4.03	0.55–0.64	[50]
Jizerské Mountains, [Jizera Mts.], Czech Rep.	mountain, isolated	2004–2007	64 males in 2005	3.37	0.46	[50]
Krušné [Ore Mts.], Czech Rep.	mountain, isolated	2004–2007	253 males in 2005	3.83–4.55	0.56–0.71	[50]
Alps, France, Switzerland, Italy	mountain, metapopulation	1997–2000	mean breeding density = 7.3 individuals/ km^2^	4.8–6.0	0.82	[51]
Finland	lowland, continous	1997–2000	mean breeding density = 7.8 individuals/km^2^	6.0–6.5	0.88	[51]
Finland	lowland, continous	–	–	—	0.74	[30]
North Germany	lowland, isolated	1994–2008	~200 birds during the study period	4.51 ± 0.20	0.54	[52]
Netherlands	lowland, decreasing		~25 birds	3.98 ± 0.24	0.49	[52]
Germany	lowland, historical	late 1960s and 1970s	~8000 birds around 1955	5.10 ± 0.16	0.69	[52]
Netherlands	lowland, historical	mainly around 1930s	~15,000 birds around 1941	4.62 ± 0.17	0.58	[52]
Denmark	lowland, historical	1905, 1938–1941, 1984	~2000 birds around 1942	3.89	0.55	[52]
Netherlands	lowland, isolated	2003–2005	17 males in 1996	3.61	0.53	[53]
Netherlands	lowland, historical	1893–1941	5000–8000 males	5.84	0.65	[53]
Norway	lowland, continuous	1991–1992	–	5.90	0.72	[53]
Alps, Austria	mountain, metapopulation	2000	–	6.35	0.73	[53]
England	lowland	2002–2003	–	4.23	0.52	[53]
Alps, Austria	mountain, metapopulation	2013–2016	150–6800 birds during the study period	3.2–3.8	0.51–0.76	[54]
Carpathians, Ukraine	mountain, isolated	2011	13,000 total Ukraine population in 2011	3.22–4.45	0.73–0.80	[55]
Northern Polisia, Ukraine	lowland	2011	13,000 total Ukraine population in 2011	3.14–3.47	0.75–0.81	[55]
Northern Sweden	lowland	1981–1983	–	3.18–3.86	0.53–0.72	[56]

## Data Availability

The original contributions presented in this study are included in the Supplementary Table S3. Further inquiries can be directed to the corresponding author.

## Supplementary Materials

The following supporting information can be downloaded at: https://www.mdpi.com/article/10.3390/genes17091038/s1. Table S1: Sampling information; Table S2: Error rate information; Table S3: Allele frequency information.
