# Supplementary material for: Genetic Differentiation Between Black Grouse (Lyrurus tetrix, Linnaeus, 1758) Populations from the Sudetes and the Carpathians
Source: Genes (Basel). 2026 Aug 29;17(9):1038. doi: 10.3390/genes17091038 (PMC13606568; doi:10.3390/genes17091038)
Supplement: Supplementary file 1 [file genes-17-01038-s001.zip › Tabela S1 sampling info31.07.pdf]

Tabel S1. Sampling information: numbers of biological samples collected at each location, grouped by year.

**Pop**	**Locality**	**Latitude (N)**	**Longitude (E)**	2009	2011	2012	2013	2014	2015	2016	2017	2018	2019	Total
**Jizera Mts**.	L1	50°51′01″N	15°25′12″E				2	10	7					19
	L2	50°50′49,20″N	15°21′51,12″E	4		1	5							10
	L3	50°50′44″N	15°29′29″E	1										1
	L4	50°49′57″N	15°24′29″E					1						1
	L5	50°53′21″N	15°16′23″E					2						2
	Total Jizera Mts.			**5**		**1**	**7**	**13**	**7**					**33**
**Giant Mts.**														
	L1	50°47′52,02″N	15°28′13,01″E			4	8	11	10	42	42	45		162
	L2	50°48′50,24″N	15°29′48,09″E							8	10	14		32
	L3	50°47′08,7″N	15°37′33,5″E							2	7	8		17
	L4	50°45′46,12″N	15°38′03,69″E				11	4	7	2				24
	L5	50°45′22″N	15°48′41″E	2			4							6
	L6	50°46′22,667″N	15°36′54,032″E							6	10	5		21
	L7	50°44′09″N	15°49′57″E				2					2		4
	L8	50°46′49,82″N	15°32′44,52″E					4						4
	L9	50°46′34,8126″N	15°36′13,8234″E					6				2		8
	L10	50°48′56,89″N	15°26′25,52″E				3					3		6
	L11	50°47′06,169″N	15°36′43,668″E									4		4
	L12	50°46′39″N	15°31′32″E									2		2
	L13	50°47′17″N	15°36′37″E									23		23
	L14	50°46′49″N	15°34′42″E									7		7
	L15	50°46′21,85″N	15°36′44,33″E									7		7
	L16	50°45′21,01″N	15°40′31,97″E								4	4		8
	L17	50°48′45,36″N	15°28′41,83″E								2	4		6
	L18	50°46′24″N	15°41′40″E								2	4		6
	Total Giant Mts.			**2**		**4**	**28**	**25**	**17**	**60**	**77**	**134**		**347**
**Tatra Mts.**														

	L1	49°12′45″N	20°04′14″E			6		6
	L2	49°13′42″N	20°03′00″E			13	2	15
	L3	49°12′55″N	20°04′06″E			1		1
	L4	49°26'22.0"N	19°51'15.0"E			6	3	9
	L5	49°12′38″N	20°04′14″E			2	8	10
	L6	49°10′38,2″N	20°07′46,0″E			3	1	4
	L7	49°13′14″N	19°45′54″E			1	4	5
	L8	49°12′45,5″N	19°50′16,3″E			10	2	12
	L9	49°14′18″N	19°57′32″E			18	9	27
	L10	49°13′52,7″N	19°57′58,1″E			1	5	6
	L11	49°14′00,1″N	19°56′24,9″E			1	1	2
	L12	: 49°14′21″N	19°59′25″E			3	1	4
	L13	49°13′49,2″N	19°58′11,2″E			1	1	2
	L14	49°14′16″N	19°56′42″E			1	1	2
	L15	49°13′06″N	19°46′31″E			4		4
	L16	49°14′49″N	19°53′12″E			3	3	6
	L17	49°14′47″N	19°52′43″E			4	4	8
	L18	49°14′26″N	19°53′22″E				1	1
	L19	49°14′12,1″N	19°45′58,9″E			9	1	10
	L20	49°13′09,7″N	19°50′01,0″E			4	9	13
	L21	49°14′55,0″N	20°00′02,3″E			5	5	10
	L22	49°14′03″N	20°03′58″E			5	5	10
	L23	49°14′40″N	20°03′10″E			1	1	2
	L24	49°11′57,7″N	19°49′11,8″E			2		2
	L25	49°12′17″N	19°50′09″E				1	1
	Total Tatra Mts.					**98**	**68**	**166**
**ONT Basin**								
	L1	49°26′35″N	19°46′42″E	18	1			19
	L2	49°27′58,363″N	19°49′20′930″E	2	2			4
	L3	49°26′00″N	19°47′05″E	5	5			10

	Total ONT Basin		**25**				**8**					**33**
**Total**		7	25	5	35	38	32	60	77	232	68	579
