# Supplementary material for: Genetic Differentiation Between Black Grouse (Lyrurus tetrix, Linnaeus, 1758) Populations from the Sudetes and the Carpathians
Source: Genes (Basel). 2026 Aug 29;17(9):1038. doi: 10.3390/genes17091038 (PMC13606568; doi:10.3390/genes17091038)
Supplement: Supplementary file 1 [file genes-17-01038-s001.zip › Table S2 ErrorRateInfo31.07.pdf]

Table S2. Genetic polymorphism, marker informativeness, and microsatellite genotyping error rates estimated from non-invasive black grouse (*Lyrurus tetrix*) samples collected in southern Poland. A – number of alleles; H_O – observed heterozygosity; H_E – expected heterozygosity under Hardy-Weinberg Equilibrium; *HWE* = significance of deviation from Hardy-Weinberg equilibrium ($P < 0.05$ indicated by *); Null – estimated frequency of null alleles at each locus calculated using the Brookfield method. ADO n – number of detected Allelic Drop-Out (ADO) events; ADO (%) – Allelic Drop-Out rate calculated as the proportion of ADO events relative to the total number of successful amplifications for a given locus. FA n – number of false allele events; FA (%) – proportion of False Alleles relative to the total number of successful amplifications for a given locus. The overall estimated genotyping error rate (ADO + FA) across all loci was **4.30%.** $P_{(ID)}$ – probability of identity; $P_{(ID\text{-}Sibs)}$ – probability of identity among siblings.

	A	H_O	H_E	*HWE*	Null	ADO n	ADO %	FA n	FA%	$P_{(ID)}$	$P_{(ID\text{-}Sibs)}$
**BG16**	9	0,730	0,782		0,035	0	0,00	0	0,00	0,072	0,377
**TTT1**	6	0,574	0,713	*	0,98	22	8,75	6	1,41	0,122	0,42
**TUT2**	3	0,172	0,215		0,76	0	0,00	2	0,65	0,628	0,799
**BG12**	14	0,803	0,898		0,054	38	13,38	0	0,00	0,019	0,306
**TUT1**	8	0,697	0,847	*	0,95	14	4,63	2	0,70	0,041	0,336
**TUT4**	10	0,631	0,683		0,036	6	2,11	1	0,70	0,154	0,466
**TUT3**	6	0,631	0,715	*	0,063	2	0,70	3	0,85	0,123	0,421
**BG18**	8	0,615	0,757	*	0,87	8	2,82	0	0,00	0,086	0,493
**BG15**	6	0,689	0,703		0,01	2	0,82	6	1,35	0,14	0,433
Overall	70	0,616	0,702			92	3,60	20	0,7	1,0E-09	4,2E-04
